# Sparring and Neurological Function in Professional Boxers

**DOI:** 10.3389/fpubh.2014.00069

**Published:** 2014-07-21

**Authors:** John W. Stiller, Steven S. Yu, Lisa A. Brenner, Patricia Langenberg, Phillip Scrofani, Patrick Pannella, Edbert B. Hsu, Darryl W. Roberts, Ray M. T. Monsell, Sidney W. Binks, Alvaro Guzman, Teodor T. Postolache

**Affiliations:** ^1^Department of Behavioral Health, St. Elizabeth’s Hospital, Washington, DC, USA; ^2^Maryland State Athletic Commission, Baltimore, MD, USA; ^3^Mood and Anxiety Program, Department of Psychiatry, University of Maryland School of Medicine, Baltimore, MD, USA; ^4^Institute of Sports Chronobiology, Washington, DC, USA; ^5^Department of Medicine, University of Southern California, Los Angeles, CA, USA; ^6^Veterans Integrated Service Network 19, Mental Illness Research Education and Clinical Center, Denver, CO, USA; ^7^Department of Psychiatry, University of Colorado, Anschutz Medical Campus, Aurora, CO, USA; ^8^Department of Physical Medicine and Rehabilitation, University of Colorado, Anschutz Medical Campus, Aurora, CO, USA; ^9^Department of Neurology, University of Colorado, Anschutz Medical Campus, Aurora, CO, USA; ^10^Department of Epidemiology and Public Health, University of Maryland School of Medicine, Baltimore, MD, USA; ^11^Institute for the Psychological Sciences, Arlington, VA, USA; ^12^Department of Emergency Medicine, Johns Hopkins University, Baltimore, MD, USA; ^13^Econometrica Inc., Bethesda, MD, USA; ^14^St. Julian’s Medical Centre, Newport, UK; ^15^Tuning, Inc., Silver Spring, MD, USA

**Keywords:** chronic traumatic brain injury, boxing, cognitive, balance, cumulative sparring index

## Abstract

Despite increased interest regarding the potentially long-term negative impact of chronic traumatic brain injury, limited research has been conducted regarding such injuries and neurological outcomes in real world settings. To increase understanding regarding the relationship between sparring (e.g., training under the tutelage of an experienced boxing coach for the purpose of improving skills and/or fitness) and neurological functioning, professional boxers (*n* = 237) who competed in Maryland between 2003 and 2008 completed measures regarding sparring exposure (Cumulative Sparring Index, CSI) and performance on tests of cognition (Symbol Digit Modalities Test, SDMT) and balance (Sharpened Romberg Test, SRT). Measures were completed prior to boxing matches. Higher scores on the CSI (increased sparring exposure) were associated with poorer performance on both tests of cognition (SDMT) and balance (SRT). A threshold effect was noted regarding performance on the SDMT, with those reporting CSI values greater than about 150 experiencing a decline in cognition. A history of frequent and/or intense sparring may pose a significant risk for developing boxing associated neurological sequelae. Implementing administration of clinically meaningful tests before bouts, such as the CSI, SDMT, and/or the SRT, as well as documentation of results into the boxer’s physicals or medical profiles may be an important step for improving boxing safety.

## Introduction

As in all other sports, the objective of a boxing match is to win. Toward this end, “hurting” the opponent is specifically intended, and injuries are sustained. As blows to the head may reduce the capacity of an opponent being able to effectively defend him or herself, or lead to injuries that could result in a fight being stopped by the referee, the head is a primary boxing target. Among professional boxers, the majority of injuries occur in the facial area (51%) (1). Additional areas of injury include the hands (17%), eyes (14%), and nose (5%) (1). Evidence from amateur and professional settings suggests that boxers may suffer from acute cognitive impairment post-injury. Areas of dysfunction noted include delayed memory, information processing and verbal fluency, and spatial and mathematical processing (2–4). Moriarity et al. also found significant slowing in simple and choice reaction time among a small group of amateur boxers (*n* = 7) whose matches were stopped by the referee (5).

Interest in the chronic consequences of professional boxing is also longstanding (1). In 1928, Martland published a seminal article titled “*Punch Drunk*” in which he hypothesized about the relationship between boxing and brain injury (6). Overtime this condition has also been called dementia pugilistica (DP), chronic traumatic brain injury (CTBI), and chronic traumatic encephalopathy (CTE) (7). According to a retrospective, randomized study by Roberts regarding CTBI among ex-boxers competing in Great Britain, approximately 17% had symptoms consistent with DP, which was believed to have been the result of repetitive concussive and/or sub-concussive head traumas, generally over the course of many years (8). Evidence also suggests that the symptoms and signs of chronic CTBI may be progressive, and become more evident toward the end of a boxer’s career or after retirement (8, 9). Such symptoms often include some combination of cognitive and/or motor dysfunction, as well as changes in mood and behavior (1, 10). Associated motor dysfunction often manifests as dysarthria, balance difficulties, Parkinsonism, and/or asymmetric pyramidal tract signs (e.g., spasticity).

“Sparring” as it relates to training in boxing involves two individuals competing in a boxing ring under the tutelage and supervision of an experienced boxing trainer or coach for the purposes of improving a boxer’s skill level and/or fitness. Sparring is performed by using various safeguards that are designed to attempt to decrease the risk of injuries as compared with actual competition. Safeguards implemented during sparring sessions may include: (1) using headgear, body protectors, and larger gloves; (2) avoiding the mismatching of boxers; (3) teaching and practicing certain boxing techniques; (4) varying the duration (minutes) of a round; (5) increasing, or decreasing, the momentum and intensity of sparring; and (6) limiting the intensity, and quantity, of exposure to head blows.

The challenge in preventing CTBI is to avoid or substantially limit the amount of damage; thereby preventing neurological impairment secondary to repetitive blows sustained in activities like sparring. In support of this ascertion, Jordan et al. reported a significant inverse relationship between the amount and intensity of sparring, and performance on selected neuropsychological tests in 42 professional boxers licensed or applying for boxing licenses (11). Specifically, scores on tests of attention, concentration, and memory appeared to be sensitive measures of the effects of sparring on selected cognitive functions. In this study, we examine the role of sparring on selected neurological functions (cognition and balance) among professional boxers. All measures were administered pre-bouts, and such are believed to be reflective of baseline functioning.

## Materials and Methods

### Participants and enrollment

This study protocol was approved by the St. Elizabeth’s Hospital Institutional Review Board (SEH IRB) and the Maryland State Athletic Commission (MSAC). Boxers competing in Maryland between 2003 and 2008 were invited to participate. During this time period, the MSAC sanctioned and regulated 95 professional boxing events in the State of Maryland (personal communication). Generally, there were multiple bouts (e.g., 6, 7) per event. The enrollment was convenience-based, depending on availability of staff, assent by the trainer and informed consent by the boxer. Those who consented were provided with the option of requesting a copy of their neurological examination, which could be used in other jurisdictions.

In the end, 237 boxers were enrolled, including 223 (94%) males and 14 (6%) females. One hundred thirty-eight (58%) self-identified as African American, 48 (20%) as Caucasian, 30 (13%) as Hispanic, 8 (3%) as Asian American, and 13 (5%) as other. All participants were between 18 and 41 years old (median = 29, range = 18–41) and had been in professional boxing for between 0 and 12 years (0 = professional debut, median = 2 years) (See Table 1 for Participant characteristics).

**Table 1:** **Participant Characteristics (*n* = 237)**.

	Mean	Standard	Median	Range
		Deviation		
Age (years)	28.5	5.2	29.0	18–41
Years of formal education	12.4	1.7	12.0	7–16
Weight at bout (pounds)	169.7	36.8	160.0	103–325
Duration of professional boxing career (years)	3.0	2.7	2.0	0–12
Number of professional bouts	9.5	10.1	6.0	0–55
Professional wins	5.6	6.4	3.0	0–30
Professional losses	3.5	5.0	1.0	0–28
Professional draws	0.5	0.8	0.0	0–4
Cumulative Sparring Index	177.2	223.1	90.0	4–1,536
Symbol Digit Modality Test (total number completed minus incorrect items)	47.5	10.4	47.0	22–79
Sharpened Romberg Test (number of trials to pass with or without sway)	1.5	1.57	1.0	0–6

To participate in the study, boxers had to be eligible to be licensed by the MSAC (see MSAC Procedures for further information) (12). To obtain a boxer’s license in Maryland, individuals must be at least 18 years of age, and those over the age of 36 must obtain “special permission” from the Commission to compete. The Commission also requires the applicant to undergo and pass a number of medical examinations including: “(1) An ophthalmological examination administered by a board-certified ophthalmologist which must be completed within 30 days prior to submitting the application; (2) a physical examination administered by a board-certified physician which must be completed within 30 days prior to submitting the application; and (3) a neurological examination administered by a board-certified neurologist or board-certified neurosurgeon which must be completed prior to the boxer participating in his or her first boxing contest during the licensing period” (12). Additional procedures are conducted at Weigh-in, which occurs on the evening before or occasionally on the morning of a scheduled event (12). Weigh-in activities generally include visits to Commission work stations: (1) Drug Testing Station; (2) Licensing Station; (3) Physician Station (pre-bout medical examination); and (4) Neurologist Station – “Boxers who do not possess a current Commission license must receive a neurological examination by the neurologist” (12). According to the MSAC Procedures: “Boxers are (also) free to volunteer to participate in the Commission’s CTBI study while spending ‘downtime’ at the boxing weigh-in activity” (12). Immediately after the match, boxers participate in a post-bout examination which is conducted using a “report form” on which the following is recorded: (1) “physical complaints voiced in a post-bout interview”; (2) “the findings of a physical examination, the type of medical suspension (i.e., technical knockout, laceration, poor conditioning, knockout, indefinite, ‘other’)”; (3) “the duration of any medical suspension”; and (4) “the nature of any injuries suffered, and recommended treatment” (12).

Boxers who volunteered to participate in the study and a witness signed a written consent (approved by the SEH IRB and the MSAC). No data gathered during study procedures were used to exclude boxers from participating in matches. Post-consent, boxers completed a self-report questionnaire regarding demographics, as well as their sparring exposure and professional and amateur record.

### Assessment tools

#### Cumulative sparring index

Self-reported sparring-related data were obtained using a procedure previously described by Jordan et al. (11) and involved calculating an individual’s CSI using self-reported information as follows: (average number of sparring sessions per week) × (average number of rounds per session) × (average intensity of the sessions) × (number of years actively training for professional boxing) (11). The average intensity of sparring was defined as 1 = minimal or no contact; 2 = moderate contact; 3 = intense, but not quite as intense as competition; 4 = as intense as actual competition (11).

#### Sharpened Romberg Test

Sharpened Romberg Test (SRT) is a sensitive, non-specific test of balance (13). An individual stood on a level surface wearing flat shoes with his or her feet aligned in a strict tandem heel-to-toe position, arms crossed over the chest, and the open palm of the hand falling on the opposite shoulder. Once stable, the boxer was asked to close his or her eyes and to attempt to maintain his/her position for 10 s. The boxer was given up to three trials to successfully maintain the tandem position for 10 s with eyes closed and the test was scored as follows:

0 = maintained station without sway on the first trial.
1 = maintained station with sway on the first trial.
2 = maintained station without sway on the second trial.
3 = maintained station with sway on the second trial.
4 = maintained station without sway on the third trial.
5 = maintained station with sway on the third trial.
6 = could not maintain station.

For the purpose of analyses a score of 0–1 = Normal. All scores higher than 1 were scored as Abnormal.

#### Symbol Digit Modalities Test

Symbol Digit Modalities Test (SDMT) is a psychometrically sound brief screening measure that is sensitive to brain damage (14–16). Performance on the SDMT is dependent on many brain functions including attention, visual scanning, and motor speed and may be considered a test of information processing speed (i.e., the capacity to complete cognitive tasks quickly and efficiently). The test can be completed in <5 min, which is ideal for the fast-paced weigh-in environment. Raw scores (total completed items minus incorrect items) were calculated and used for statistical analyses.

#### Statistical analysis

Descriptive analyses included means, medians, and *t*-tests for continuous data, including the SDMT; chi-square tests for categorical data, including the SRT, categorized as “abnormal” (scores 2–6) or “normal” (scores 0–1).

The CSI was log-transformed for most analyses because it was highly positively skewed. Pearson correlations were calculated to assess associations among continuous variables; *t*-tests and chi-square tests compared patients identified as “normal” or “abnormal” on the SRT. A Loess (smoothing) curve of SDMT by logCSI was plotted. Both linear regression and linear spline regression were used to estimate relationships between logCSI scores on the SDMT, and adjusted for demographic variables. Logistic regression analyses were used to estimate adjusted odds ratios (OR) for normal vs. abnormal SRT by logCSI.

## Results

**Figure 1:**
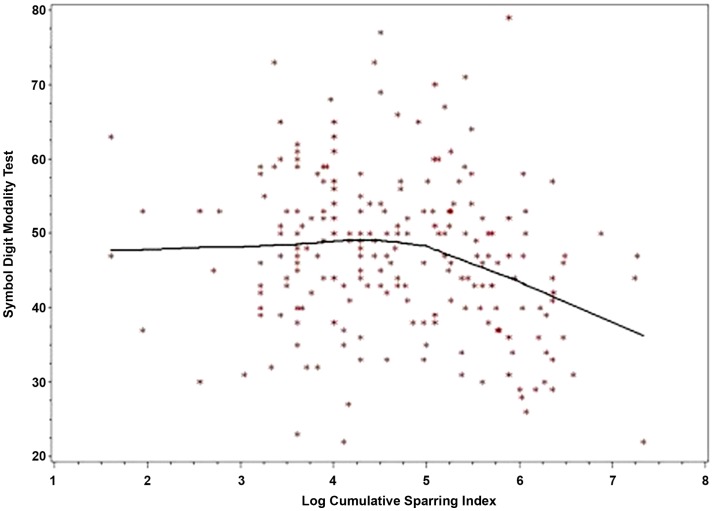
**Symbol digit modality by log cumulative sparring index with loess fit**.

LogCSI was inversely related to performance on the SDMT in the linear regression model *p* < 0.004 (Table 2, Model 1). Using linear spline regression with change-point set at five as indicated by the Loess smoother, there was little, if any, association at lower values of CSI (CSI < 148, logCSI < 5) but a strong negative association at higher CSI (CSI ≥ 148, logCSI ≥ 5), *p* = 0.0001 (Table 2, Model 2). A LOESS plot of SDMT by logCSI illustrates this threshold effect (Figure 1). In the range of the negative correlation, an increase in logCSI of 1 was associated with a decline in SDMT of about six points.

**Table 2:** **Linear (Model 1) and Spline Regression (Model 2 – for Cutpoint at Log Sparring Index = 5) for Symbol Digit Modality Test by Log Sparring Index (LogCSI) Adjusted for Age (Model 1) and Gender, Age, and Education (Model 2)**.

Effect	Coefficient	Standard	*p*-Value
		Error	
**MODEL 1**			
LogCSI	−1.809	0.620	0.004
Age	−0.066	0.131	0.62
**MODEL 2 (SPLINE)**			
LogCSI
≤5	0.72	1.03	0.49
>5	−6.18	1.59	0.0001
Age (10 years older)^a^	−0.84	1.29	0.51
Gender male	−5.22	3.01	0.08
Education > high school	5.84	1.4	<0.0001

*^a^ Coefficient represents the effect that 10 years older in age would have (e.g., 30 vs. 20, or 35 vs. 25)*.

The inverse relationship between the CSI and performance on the SRT was significant (*p* = 0.01) in a logistic regression model assessing the relationship between logCSI and scoring “abnormal” vs. “normal” on the SRT adjusted for age. A one-unit increase in log (CSI) was associated with 1.42 times (42%) higher odds of scoring abnormal on the SRT. No threshold effect was identified.

Although female boxers performed better on both tests compared to males, five points higher on SDMT and 14% abnormal females vs. 38% abnormal males on SRT, there were insufficient female subjects (*n* = 14) for a conclusive subanalysis.

## Discussion

Findings suggest a significant negative association between the amount and intensity of sparring and performance on both measures. That is, the greater the sparring exposure, the poorer the performance on both tests of information processing speed and balance. In particular, the strong association between sparring exposure and poorer performance on the SDMT is consistent with the previous report by Jordan et al. (11). Although this association, as well as the association with poorer performance on a test of balance does not prove causation, it is highly suggestive and consistent with what many involved in the sport of boxing have suspected. Specifically, exposure to repetitive sub-concussive trauma from frequent and intense sparring appears to be a significant risk for developing neurological sequelae associated with professional boxing.

A threshold effect observed for SDMT was consistent with what has been described clinically, that the onset of obvious deficits tends to occur late in a boxer’s career or after retirement (8). One possible explanation for this is that there are compensatory mechanisms that allow a boxer to perform at the same level on testing, despite underlying brain damage. However, once a certain threshold is reached, such mechanisms begin to fail and cognitive deterioration becomes apparent. Interestingly, in other populations affected by cognitive impairment [e.g., those with Multiple Sclerosis (MS)], scores on the SDMT have been shown to be relatively independent from proxies of cognitive reserve, and therefore hypothesized to be the “most robust correlate of brain pathology” (p. 3) (17). Additional research is required to explore the relationship between scores on the SDMT and proxies of cognitive reserve (e.g., premorbid IQ) among those with CTBI.

As indicated above, no threshold effect was noted with the SRT (balance). However, because the SRT data were based on a 0–6 scale, it would be difficult to observe a threshold effect.

Within the boxing community itself, there are notable variations in sparring practices. These variations include the number of sessions per week, intensity, rounds, precautionary measures, weight differentials, and skill levels between fighters. Although boxing gyms may have informal rules or “codes of conduct” regarding sparring, it is usually the professional boxer’s trainer (or coach) who ultimately determines the sparring practices for an individual boxer. There is a wide variation in the knowledge and experience of those who work as trainers. Experienced trainers generally limit the amount of moderate to high intensity sparring and spend more time on teaching/practicing techniques (i.e., balance, foot work, defensive skills, bag work, combination punching, etc.). More intense levels of sparring are generally a few days apart to allow for recovery, and sessions are often designed to build in intensity with successive rounds, and capped at a reasonable amount (e.g., no more than 12 3-min rounds in a single session). However, some trainers have the mindset that “the more sparring the better,” thereby further increasing exposure. Knowledgeable boxing trainers require their fighters to wear appropriate protective headgear, mouth guards, and use larger gloves (e.g., 16–18 oz) for maximal protection (18). Unfortunately, some trainers and/or the boxers may be less inclined to follow such strict safety rules when sparring. Some gyms may have less than optimal equipment including poorly constructed headgear and only 12–14 oz gloves available, which may result in a significant difference in the cumulative exposure to neurotrauma during sparring. Finally, poorly supervised mismatching of boxers with either weight or skill discrepancies can lead to unnecessary and excessive neurotrauma. It follows that a knowledgeable and ethical trainer is in a position to significantly reduce the risks associated with sparring. This suggests that an educational program (perhaps including an apprenticeship) leading to a meaningful licensure of trainers may have a positive effect on reducing boxing associated brain injuries.

Limitations to this study include the lack of age matched controls who are not involved in collision sports, the convenience sampling, and the cross-sectional nature of the study design. There are certain inherent limitations to information captured in a real world setting, and to the assessment tools selected for study, which could feasibly be administered in this environment.

Although the actual existence of CTBI/CTE as distinct entity has been questioned (19), the essential neuropathological features of what had previously been called Dementia Pugilistica in ex-boxers (and is now usually referred to as CTE) had been described in the 1970s (20). Recently, an unprecedented interest in CTBI/CTE was triggered by case reports of CTE in well-known ex-NFL players (21, 22), as well as neuroimaging studies in aging NFL players (23). Beyond boxing, repetitive exposure to concussive and/or subconcussive impacts and the development of CTBI/CTE became a central concern in particular for athletes participating in collision sports (24), military personnel and veterans (25). Our reported relationship between cumulative exposure to neurotrauma and impaired performance on SDMT is of particular conceptual interest considering the possible association between well-defined degenerative dementias and traumatic brain injury (26, 27). Since certain individuals could be more vulnerable to develop dementia in response to neurotrauma (28) it will be important to pursue, ideally longitudinally, molecular and neurophysiological markers that could moderate or mediate predictive associations between repetitive subconcussive head blows and neurocognitive dysfunction. This will lead to a better understanding of the natural history of CTBI/CTE, its risk and protective factors, and, perhaps, point towards novel interventions to prevent or minimize neuropathology and functional impairment.

While the ultimate goal in this long-term project is to analyze prospectively and define clinical indicators that may delineate a period of time (or “window of opportunity”) during which a boxer (or other at risk individual) could avoid or reduce the chance of developing CTBI by avoiding further exposure, this initial report suggests avoiding overzealous sparring exposure may lead to less long-term neurological dysfunction in professional boxers. In addition, the findings suggest that measures such as the SDMT (information processing speed) and SRT (balance) may be indicators of cognitive and neurological changes associated with repetitive, sub-concussive blows. Incorporating pre-bout administration and results from these simple, yet clinically meaningful tests into the boxer’s physical and/or medical profiles may be an important step for improving boxing safety.

## Conflict of Interest Statement

The authors declare that the research was conducted in the absence of any commercial or financial relationships that could be construed as a potential conflict of interest.
